# Development of a real-time RT-PCR assay for the detection of pan-human parechoviruses

**DOI:** 10.1186/s12985-021-01689-8

**Published:** 2021-11-20

**Authors:** Huanhuan Lu, Jinbo Xiao, Keyi Zhang, Zhenzhi Han, Yang Song, Dongyan Wang, Tianjiao Ji, Dongmei Yan, Shuangli Zhu, Wenbo Xu, Yong Zhang

**Affiliations:** 1 WHO WPRO Regional Polio Reference Laboratory and National Health Commission Key Laboratory of Biosafety, National Health Commission Key Laboratory of Medical Virology, National Institute for Viral Disease Control and Prevention, Chinese Center for Disease Control and Prevention, Beijing, People’s Republic of China; 2 Center for Biosafety Mega-Science, Chinese Academy of Sciences, Wuhan, 430071 People’s Republic of China

**Keywords:** Human parechoviruses, *VP1* region, Real-time RT-PCR, Typing, Phylogenetics

## Abstract

**Background:**

*Parechoviruses* (PeV-As), which constitute a new genus within the family *Picornaviridae*, have been associated with numerous localized outbreaks of serious diseases, such as coryza, pneumonia, maculopapular exanthem, and conjunctivitis. However, to the best of our knowledge, only a few laboratories worldwide conduct tests for the identification of this group of viruses. Therefore, in this study, we aimed to develop and validate a real-time RT-PCR assay for the identification of PeV-As.

**Methods:**

To design and validate a real-time PCR primer–probe targeting the *5′-UTR* region of PeV-As, the 5′-*UTR* sequences of PeV-As available in GenBank were aligned using the MUSCLE algorithm in MEGA v7.0. Thereafter, the highly conserved 5′-*UTR* region was selected, and its primer–probe sequence was designed using Primer Premier v5.0. This primer–probe sequence was then evaluated for specificity, sensitivity, and repeatability, and for its validation, it was tested using fecal samples from 728 healthy children living in Beijing (China).

**Results:**

The PeV-A real-time RT-PCR assay detected only the RNA-positive standards of PeV-A genotypes (1–8, 14, 17, and 18), whereas 72 serotypes of non-PeV-A EV viruses were undetected. In addition, the *VP1* region of these 11 PeV-A genotypes that tested positive were amplified using the primers designed in this study. Typing results indicated that eight, one, and two strains of the 11 were PeV-A1, PeV-A4, and PeV-A6, respectively. We also determined and presented the genetic characterization and phylogenetic analyses results corresponding to these 11 *VP1* region sequences. Furthermore, real-time RT-PCR assay showed good sensitivity with *LOD* of 10^2^ copies/μL. Positive results in eight parallel experiments at each concentration gradient from 10^7^ copies/μL to 10^2^ copies/μL, indicating good repeatability.

**Conclusion:**

Our findings suggested that the real-time RT-PCR assay developed in this study can be applied for routine PeV-A identification. We detected PeV-A1, 4 and 6 genotypes in the 728 faecal samples using this method. Additionally, we believe that our results will serve as a foundation for further studies on PeV-As and facilitate the expansion of the gene sequence information available in GenBank.

## Background

Human parechoviruses (PeV-As), which are RNA viruses that are closely related to enteroviruses of the family *Picornaviridae,* were first isolated several decades ago [1]. Of the 19 parechovirus genotypes (PeV-A1–19) that are currently known to infect humans, genotypes 1 and 3 are most commonly associated with diseases [2, 3]. It has also been observed that PeV-As, which commonly cause high persistent fevers in infants aged less than 5 months, are associated with sepsis-like conditions. Reportedly, the outbreak of a sepsis-like disease in children that occurred in Australia between 2013 and 2014 was caused by PeV-A3 [4]. It is also known that maculopapular exanthema, which is a common and distinctive rash involving the extremities, with palmar and plantar erythema is associated with PeV-A3 infection (Table 1). To effectively treat such PeV-A-associated diseases, the development of a sensitive and reproducible PeV-A identification method with good specificity is necessary.

**Table 1:** Classification and clinical symptoms of PeV-A

Genotype of PeV-A	Clinical symptoms
PeV-A1	Mainly gastrointestinal tract symptoms (diarrhea and emesis) and respiratory tract symptoms (cough, wheezing, rhinorrhea, tachypnea and herpangina), followed by fever, rash, meningitis, encephalitis and acute flaccid paralysis
PeV-A3	Mainly neonatal fever, meningitis, encephalitis and neonatal sepsis-like disease, followed by gastrointestinal tract symptoms (diarrhea and emesis), respiratory tract symptoms (cough, wheezing, rhinorrhea, tachypnea and herpangina), rash and acute flaccid paralysis
PeV-A4	Mainly gastrointestinal tract symptoms (diarrhea and emesis) and respiratory tract symptoms (cough, wheezing, rhinorrhea, tachypnea and herpangina), followed by Neonatal fever, meningitis and encephalitis
PeV-A6	Gastrointestinal tract symptoms (diarrhea and emesis), respiratory tract symptoms (cough, wheezing, rhinorrhea, tachypnea and herpangina)
PeV-A2, 5, 8, 10	Gastrointestinal tract symptoms (diarrhea and emesis)
PeV-A7, 9, 11–19	Unknown

PeV-As are positive-strand RNA viruses belonging to the genus *Parechovirus*, which is part of the expanding *Picornaviridae* family. The PeV-A genome is approximately 7300 bp long and divided into four distinct regions, namely, the 5′-untranslated region (*5′-UTR*), an open reading frame region (*ORF*), the *3′-UTR* region, and the poly(A) tail region, which is similar to the enterovirus genome [5, 6]. Additionally, PeV-A capsids each comprise 60 copies of three viral proteins, *VP0*, *VP1*, and *VP3* [7], that cleave post-translationally from a single polyprotein that forms the basic building block of the capsids. Moreover, in most small RNA viruses, *VP0* is cleaved into fragments *VP2* and *VP4* following viral particle assembly [8]. Nonetheless, unlike the VP0 of other small-RNA viruses, the *VP0* of PeV-As remains intact, resulting in this capsid containing only three instead of four distinct polypeptides [7, 9]. Specifically, human parechoviruses type 1 and 2 (PeV-A1 and PeV-A2, respectively), which were first identified in 1956 during a summer outbreak of diarrhea in children, were classified as echoviruses type 22 and 23 in the genus *Enterovirus* (EV), respectively [10, 11]. The roles played by these pathogens in human diseases were recently elucidated via molecular diagnostic techniques. In 1997, advances in molecular biology led to the discovery of significant differences between the genomic structures, encoded proteins, and biological properties of these pathogens and those of enteroviruses, resulting in them being renamed parechoviruses type 1 and 2, respectively [12]. The genome length of the structural protein-coding region of PeV-A is shorter than that of EV, and the genome length of the non-structural protein-coding region is longer than that of EV. Some of the PeV-A types (PeV-A1-A2, A4-A6) contain arginine-glycine-glutamate amino acids (RGD) at the carboxyl terminus of *VP1*. Among the genus *Parechovirus*, only PeV-A can infect human. Although the *5'-UTR* region is conserved in PeV-A, it is distinct from other viruses of *picornaviridae*, therefore, primers with *5'-UTR* region can specifically detect PeV-A. Moreover, similar to enteroviruses, PeV-As, which primarily replicate in the gut, are typically transmitted via the fecal–oral route but can also be transmitted via the nasopharyngeal route [13]. The slow, laborious, and insensitive cell culturing processes that are used to diagnose PeV-A infections have been largely replaced by nucleic acid amplification procedures such as reverse transcription polymerase chain reaction (RT-PCR), which can be used to target the conserved *5′-UTR* region [5–9]. Furthermore, in 1999, a molecular typing method based on aligning the RT–PCR-amplified sequences of the *VP1* coding region with sequence data corresponding to all PeV-A genotypes (the nucleotide sequence consistency in the *VP1* region of identical strains was greater than 75%) was introduced as a more effective technique for genotype determination [14, 15]. In summary, for the detection of PeV-A, real-time RT-PCR with 5'-UTR region probe-primer is usually used to determine the negative or positive of the samples, and RT-PCR with VP1 region primers is used to further determine the genotype of the positive sample, so as to better understand the epidemiological characteristics of PeV-A.

Therefore, in this study, we developed and validated a real-time RT-PCR assay for the identification of PeV-As using 728 clinical samples. Positive samples were amplified via nested RT-PCR, and the *VP1* region was sequenced using the primers designed in this study. We also performed characterization and phylogenetic analysis on the *VP1* sequence. The epidemiological characteristics of PeV-A in China were further analyzed through genotyping of the positive samples. The results of this study provide a firm theoretical basis for further studies on PeV-As and help to expand the gene sequence information available in GenBank.

## Methods

### Real-time RT-PCR

#### Primer and probe design

The *5′-UTR* sequences of PeV-As available in GenBank were aligned using the MUSCLE algorithm in MEGA v7.0 [16]. Based on the results of this multiple sequence alignment, the highly conserved *5′-UTR* region was selected, and the *5′-UTR* primer–probe sequence (Table 2) was designed in accordance with the principles for designing TaqMan RT-PCR primer and probe sequences using Primer Premier v5.0 (Premier Biosoft, Palo Alto, CA, USA). In addition, the designed primer and probe sequences were screened via BLAST to detect the possibility of cross-reactivity with non-PeV-As.

**Table 2:** Forward (F) and reverse (R) primers and probe (P) for the detection of human parechovirus (PeV-A) and primers for amplification of the *VP1* region of PeV-A

Name	Primer sequence (5′–3′)	Nucleotide Position (nt)*	References
PeV-A-*5’UTR*-F	TGACGTGTGGGAACTTRTTGG	352–372	This study
PeV-A-*5’UTR*-R	CCTTCGTGGGCCYTACAACTAGTG	538–561	This study
PeV-A-*5’UTR*-P	CYCTGGGGCCAAAAGCCAAGGTTT	433–456	This study
PeV-A-*VP1*-F1	TTYTCYACTTGGATGMGGAARA	2159–2180	This study
PeV-A-*VP1*-R1	ATRCTYGACATHARNCCWGC	3392–3411	This study
PeV-A-*VP1*-F2	CNTGGGGYTCVCARATGGAYYT	2340–2361	This study
PeV-A-*VP1*-R2	CCATARTGYTTRTARAAVCCYCTRT	3081–3105	This study

^*^ Relative to the Harris PeV-A1 strain

#### Preparation of positive standards

Only 11 PeV-A genotypes (1–8, 14, 17, and 18) with *5′-UTR* region sequences were available in the GenBank database. Thus, the *5′-UTR* regions of these strains, PeV-A1 (*Harris strain*), PeV-A2 (*Williamson strain*), PeV-A3 (*A308/99 strain*), PeV-A4 (*K251176-02 strain*), PeV-A5 (*CT86-6760 strain*), PeV-A6 (*NII561-2000 strain*), PeV-A7 (*PAK5045 strain*), PeV-A8 (*BR/217/2006 strain*), PeV-A14 (*V3C strain*), PeV-A17 (*M36/CI/2014 strain),* and PeV-A18 (*GhanaA36 886 strain*), were used as target sequences. After their synthesis, they were cloned into the p-GEM-T Easy Vector system (Promega, Madison, WI, USA). Thereafter, the plasmids containing these target sequences were cloned by TsingKe Biological Technology Limited (Beijing, China). Additionally, the plasmids were linearized using restriction endonuclease *Sac I,* and DNA purification was performed using AMPure XP (Beckman Coulter, CA, USA). The purified linear plasmids were transcribed in vitro using a RiboMAX™ Large Scale RNA Production System-T7 kit (Promega, Hilden, Germany) and thereafter purified using the RNeasy Mini Kit (Promega, Hilden, Germany). To calculate the copy numbers, the purified RNA was then spectrophometrically quantified using the Qubit™ RNA BR Assay Kit (Invitrogen, Eugene, OR, USA).

#### Reagents, reaction systems, and real-time RT-PCR settings

As a template, 3-μL samples of the nucleic acids were used (the total reaction volume was 25 μL) in a One Step PrimeScript™ RT-PCR Kit (Perfect Real Time, TaKaRa, Dalian, China). The concentrations of each primer and probe in the reaction mixtures were 0.8 and 0.4 μM, respectively. The reaction settings were as follows: the system was maintained at 42 °C for 10 min and 95 °C for 1 min. This was followed by40 cycles of amplification at 95 °C for 10 s and 55 °C for 40 s. All the real-time RT-PCR experiments were performed using QuantStudio 5 instruments (Applied Biosystems, Thermo Fisher Scientific, Waltham, MA, USA).

#### Specificity testing

To assess the specificity of the PeV-A assay, 72 known non-PeV-A strains, including 16 serotypes of EV-A (CVA2–A8, CVA10, CVA12, CVA14, CVA16, EV-A71, EV-A76, EV-A89, EV-A90, and EV-A120), 45 serotypes of EV-B (CVB1–B6, CVA9, E1–7, E9, E11–21, E24–27, E29–33, EV-B74–B75, EV-B79–B81, EV-B83, EV-B85, EV-B93, EV-B97, and EV-B106), and 11 serotypes of EV-C (PV-1, PV-3, CVA1, CVA11, CVA13, CVA17, CVA20–A21, CVA24, EV-C96, and EV-C99) were tested together using 11 types of PeV-A (1–8, 14, 17, and 18) RNA-positive standards. Enterovirus-specific real-time RT-PCR assays showed that all the non-PeV-A strains were positive. Thereafter, they were stored in our laboratory. Viral RNA was extracted using a QIAamp Viral RNA Mini Kit (Qiagen, Hilden, Germany), in accordance with the manufacturer’s instructions.

#### Sensitivity and stability testing

Globally, the most common PeV-A types are PeV-A1 and PeV-A3. Therefore, the sensitivity and stability of the probes was evaluated based on these two PeV-As. The limit of detection (LOD) of the real-time RT-PCR assay was evaluated via a serial tenfold dilution of PeV-A1 (2.1 × 10^8^–2.1 × 10^1^ copies/μL) and PeV-A3 (1.9 × 10^8^–1.9 × 10^1^ copies/μL). The results were interpreted as ‘positive’ for threshold cycle (Ct) values ≤ 35 and ‘negative’ for Ct > 35. The obtained PeV-A1 and PeV-A3 RNA standards were diluted tenfold. In this regard, the approximate proportion of the Ct value that could be explained by the copy number was determined based on the coefficient of determination (*R*^2^). Thereafter, seven different concentrations (2.1 × 10^7^–2.1 × 10^1^ and 1.9 × 10^7^–1.9 × 10^1^ copies/μL for PeV-A1 and PeV-A3, respectively) were selected as templates based on eight parallel replicate experiments.

### Clinical samples for validation

#### Sample material

To further validate this method, experiments were conducted using stool from healthy children in Beijing. This study involved no human experimentation. Within the June–November 2019 period, a total of 728 stool samples were collected from 549 healthy children living in Beijing, China. All the 549 children, who participated in the study, were aged below 5 years. Of these 549 children, 284 (51.73%) were boys, and 265 (48.29%) were girls. The stool samples were collected from children living in five districts in Beijing (Haidian, n = 179; Daxing, n = 183; Dongcheng, n = 117; Huairou, n = 122; and Fengtai, n = 127). These samples were sent to the National Institute for Viral Disease Control and Prevention, Chinese Center for Disease Control and Prevention (CDC) for the investigation on enterovirus prevalence in healthy population.

#### Stool sample treatment and nucleic acid extraction

All the fecal samples were intended for nucleic acid extraction; therefore, they required special handling. The stool samples (approximately 2 g each) were dissolved in 10 mL of phosphate-buffered saline containing antibiotics and thereafter treated with 1 g of glass beads and 1 mL of chloroform, followed by vigorous shaking for 20 min using a mechanical shaker and centrifugation for 20 min at 1500 × *g* in a refrigerated centrifuge. Then, the supernatant was stored at − 80 °C until further analysis. RNA was extracted from the stool suspension using a QIAamp Viral RNA Mini Kit (Qiagen, Hilden, Germany).

#### Comparison of different real-time RT-PCR assays for PeV-A detection

A TaKaRa One Step PrimeScript™ RT-PCR Kit (Perfect Real Time) was used to detect viral RNA via real-time RT-PCR using the probe/primer sequences of PeV-A designed in this study and the probe/primer sequences of PeV-A reported in a previous study (AN345, AN344, and AN257) [17]. The reaction settings corresponding to the probe/primer sequences designed in this study were as follows: the system was maintained at 42 °C for 10 min and at 95 °C for 1 min, followed by two 40 cycles of amplification at 95 °C for 10 s and at 55 °C for 40 s. The reaction settings corresponding to primer/probe sequences, AN345, AN344, and AN257, for PeV-A detection were set as previously reported [17].

#### *VP1* region amplification and sequencing and molecular typing

The *VP1* region of PeV-As was amplified via nested PCR using the Primescript One Step RT-PCR Kit Ver.2 (TaKaRa). The first reaction system (25 μL) contained 3 μL of the original sample template RNA, 1 μL of PrimeScript 1 Step Enzyme Mix, 12.5 μL of 2 $\times$ 1 Step Buffer (Dye Plus), 7.5 μL of RNase Free dH_2_O, 0.5 μL of forward primer-F1, and 0.5 μL of reverse primer-R1 (all the final concentrations were 20 μM). The reaction settings were as follows: the system was maintained at 50 °C for 30 min and at 94 °C for 2 min, followed by 40 cycles of amplification at 94 °C for 30 s, 50 °C for 30 s, 72 °C for 1 min 20 s, and finally 72 °C for 7 min. In the second reaction system, the template was the product of the previous reaction, and the primers were F2/R2 (Table 2). The reaction settings were as follows: the system was maintained at 94 °C for 2 min, followed by 40 cycles of amplification at 94 °C for 30 s, 50 °C for 30 s, 72 °C for 1 min 20 s, and finally 72 °C for 7 min. The PCR product was purified using a QIAquick PCR purification kit (Qiagen, Hilden, Germany) and sequenced in both directions using an ABI 3130 Genetic Analyzer (Applied Biosystems, Foster City, CA, USA), resulting in each strand being sequenced at least once. The EV Genotyping Tool and BLAST were used to identify the parechovirus type, based on the obtained partial *VP1* sequences [18].

#### Phylogenetic analysis

The *VP1* sequences of the PeV-A strains were aligned with PeV-A prototype strains using the MUSCLE algorithm implemented via MEGA v7.0 [16]. A nucleotide identity matrix and an amino acid identity matrix were generated using BioEdit v7.0.9.0 [19]. Eleven strains as well as the nucleotide and amino acid sequences that showed more than 90% similarity to those of the *VP1* region, based on BLAST, were downloaded to construct a maximum likelihood (ML) tree. The ML trees constructed using the GTR + G model, as suggested by ModelGenerator0.85, were then implemented in MEGA with 1000 bootstrap replicates. In addition, to verify the best topology, ML trees were constructed using RAxML version8.2.12 [20].

## Results

### Real-time RT-PCR

#### Specificity testing

**Fig. 1:**
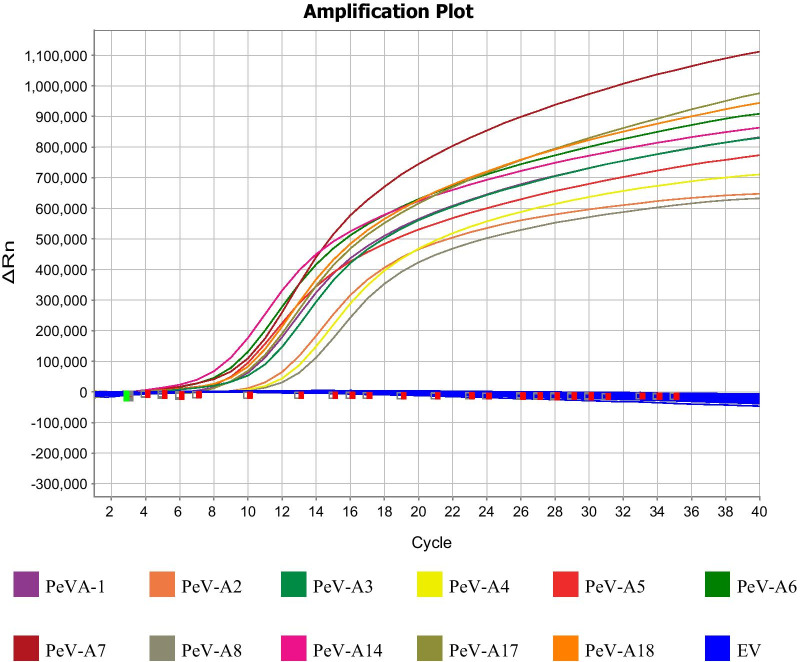
Evaluation of the specificity of real-time RT-PCR assay for PeV-A detection

In this study, performing the real-time RT-PCR assay for the identification of PeV-As led to the detection of only the RNA-positive standards of PeV-A genotypes (1–8, 14, 17, and 18), while 72 serotypes of non-PeV-A EV viruses were undetected (Fig. 1).

#### Sensitivity testing

**Fig. 2:**
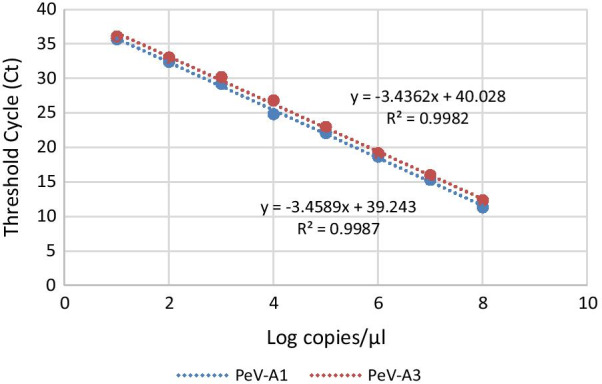
Dynamic range and linearity of PeV-A real-time assay. Threshold cycle (Ct) values (*y*-axis) were plotted against tenfold dilutions of PeV-A1 and PeV-A3. R^2^, regression coefficient

To determine the sensitivity of the PeV-A real-time RT-PCR assay, serial dilution of RNA transcripts derived from the clones of the *5′-UTR* region of the 11 PeV-A genotypes (1–8, 14, 17, and 18) was performed, and the corresponding Ct values were recorded (Fig. 2). Thereafter, standard curves were constructed using the Log value of the RNA copies as the x-axis and the Ct value as the y-axis. The coefficient of determination (*R*^*2*^) values were 0.9987 and 0.9982, respectively, and the LODs were both 10^2^ copies/μL.

#### Repeatability testing

To determine the repeatability of the real-time RT-PCR assay, seven RNA-positive standards with different PeV-A1 and PeV-A3 concentrations (2.1 × 10^7^–2.1 × 10^1^ copies/μL and 1.9 × 10^7^–1.9 × 10^1^ copies/μL, respectively) were tested using eight parallel replicates. Positive results in eight parallel experiments at each concentration gradient from 10^7^ copies/μL to 10^2^ copies/μL (Table 3), indicating good repeatability.

**Table 3:** The reproducibility of the PeV-A real-time RT- PCR

Copies/reaction	No. of positive samples/no. of samples test by real-time RT-PCR for detection of PeV-A1 and PeV-A3^a^	
	PeV-A1	PeV-A3
10^7^ copies/μL	8/8	8/8
10^6^ copies/μL	8/8	8/8
10^5^ copies/μL	8/8	8/8
10^4^ copies/μL	8/8	8/8
10^3^ copies/μL	8/8	8/8
10^2^ copies/μL	8/8	8/8
10^1^ copies/μL	0/8	0/8

^a^The results were interpreted as ‘positive’ for threshold cycle (Ct) values ≤ 35 and ‘negative’ for Ct > 35

### Validation of clinical specimens

#### Virus detection and genotyping

Using the probe-primer designed in this study, 11 samples tested positive for PeV-A, eight of which were positive with the probe-primers, AN345, AN344, and AN 257. The prevalence of PeV-A infection did not differ significantly between boys and girls (*P* > 0.1). Additionally, the partial *VP1* region (696–702 bp) of the 11 strains was successfully determined via RT-PCR, and three types of PeV-As, including PeV-A1 (n = 8), PeV-A4 (n = 1), and PeV-A6 (n = 2), were identified using the Online Enterovirus Genotyping Tool (Table 4).

**Table 4:** Comparison of two methods used to test 728 fecal samples

Virus type	Probe-primer	No. positive^a^	Genotype (no.)^b^	References
PeV-A	PeV-A-5’UTR-F/R/P	11	PeV-A1 (n = 8), PeV-A4 (n = 1), PeV-A6 (n = 2)	This study
	AN344, AN345, AN257	8	PeV-A1 (n = 7), PeV-A6 (n = 1)	[17]

^a^Results of real-time RT-PCR test

^b^Result of molecular typing detected via RT-PCR

#### Genomic characterization of *VP1* region

The nucleotide sequences of the 11 strains isolated from the stool samples were compared pairwise with those corresponding to the PeV-A1 (*Harris strain*), PeV-A4 (*K251176-02 strain*), and PeV-A6 (*NII561-2000 strain*) prototype strains, as well as other PeV-A prototype strains. Similarities between the nucleotide sequences of the *VP1* region and the amino acid sequences of eight PeV-A1 strains and the PeV-A1 prototype strain were 73.9–75.4% and 87.9–89.2%, respectively. Additionally, the similarities between the nucleotide sequences of the *VP1* region and amino acid sequences of the PeV-A4 strain and PeV-A4 prototype strain were 82.6% and 95.6%, respectively, and those between the nucleotide sequences of the *VP1* region and amino acid sequences of the two PeV-A6 strains and the PeV-A6 prototype strain were 94.7–95.6% and 96.9%, respectively.

#### Phylogenetic analysis of the *VP1* region

**Fig. 3:**
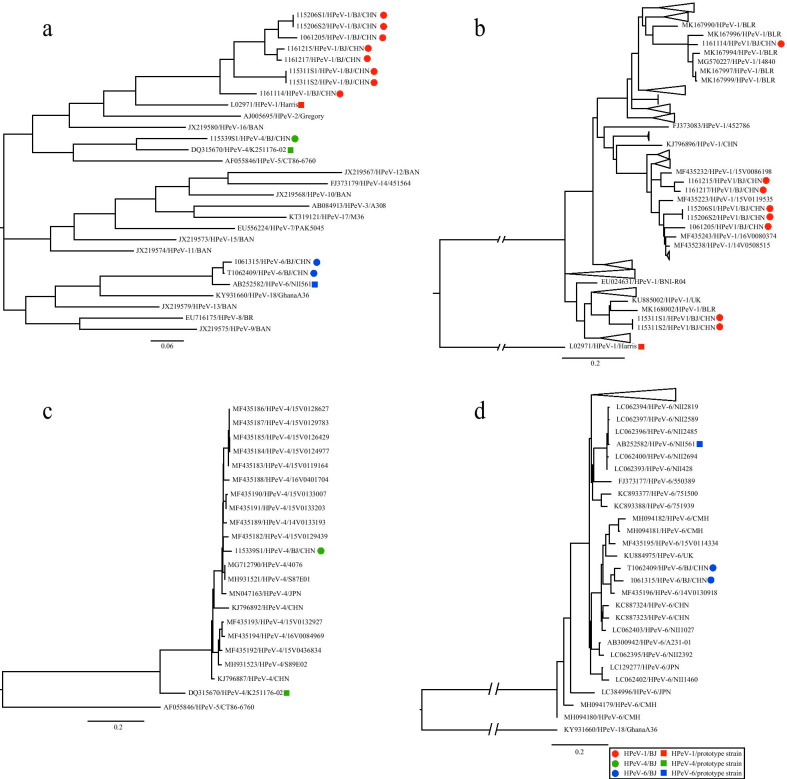
Phylogenetic tree based on the sequences of the *VP1* region of PeV-As. Maximum likelihood trees were constructed using the GTR + G model and implemented in MEGA7.0 with 1000 bootstrap replicates. The circles and squares represent the 11 detected PeV-A strains and the PeV-A prototype, respectively. The scale bars indicate the genetic distance. All panels have the same proportions. **a** Eleven PeV-A strains and PeV-A prototype strains, **b** Eight PeV-A1 strains and PeV-A1 sequences downloaded from BLAST server, **c** One PeV-A4 strain and PeV-A4 sequences downloaded from BLAST server, **d** Two PeV-A6 strains and PeV-A6 sequences downloaded from BLAST server. *// Human parechovirus (PeV-A) truncated to a quarter of its original size for better clarity. * A clearer presentation of the results of this study; a part of the sequence is hidden using a triangle

A phylogenetic tree was established based on the nucleotide sequences of the *VP1* coding regions of the 11 PeV-A strains identified and all the PeV-A prototype strains in the GenBank database (Fig. 3a). This phylogenetic tree indicated that eight strains clustered with the PeV-A1 prototype strain, while one strain clustered with the PeV-A4 prototype strain, and two strains clustered with the PeV-A6 prototype strain, confirming the molecular typing results.

The phylogenetic tree based on the nucleotide sequences downloaded from BLAST and eight *VP1* region sequences showed that three of the PeV-A1 strains clustered with strains isolated from Belarus, while the remaining five PeV-A1 strains clustered with strains isolated from Hong Kong (Fig. 3b). Moreover, the phylogenetic tree based on the PeV-A4 strains showed that the PeV-A4 strain clustered with strains isolated from Hong Kong (Fig. 3c), while the two PeV-A6 strains clustered with strains isolated from Hong Kong (Fig. 3d).

## Discussion

*Parechoviruses* constitutes a new genus within the *Picornaviridae* family, and PeV-A1, which is considered to be a very common pathogen, is prevalent worldwide [2]. PeV-A3 has caused several global outbreaks of severe diseases in children [3, 4, 21]. Even though parechoviruses and enteroviruses are similar in terms of genomic structure [1], enterovirus testing methods do not detect parechoviruses. Thus, molecular diagnostic methods, such as real-time RT-PCR, are becoming increasingly important in disease diagnosis owing to their rapid sensitivity. Furthermore, sensitive nucleotide detection methodologies contribute to a better understanding of the epidemiology and natural history of diseases. Therefore, in this study, we designed and validated a real-time RT-PCR primer–probe targeting the *5′-UTR* region of PeV-A. PeV-A-positive standards were tested together with 72 serotypes of known non-PeV-A strains (enteroviruses), and the results showed no cross-reactions and specifically detected only PeV-As. The LOD of this real-time PCR assay was 10^2^ copies/μL, indicating that the assay system has good sensitivity. Additionally, positive results in eight parallel experiments at each concentration gradient from 10^7^ copies/μL to 10^2^ copies/μL, indicating good repeatability. Given that the *5′-UTR* regions of different types of PeV-As are highly conserved, it is possible that this assay has potential for application in detecting new or emerging PeV-As. The commercial pan-parechovirus techniques, namely: Biomérieux's Biofire (meningo-encephalitis panel) and Fast Track respiratory diagnosis (FTD 33) have their own advantages. Biomérieux's Biofire (meningo-encephalitis panel) has good sensitivity, with 95% detection limits in CSF of 0.62 TCID_50_/mL (PeV-A1) and 9.92 TCID_50_/mL (PeV-A) using NucliSENS® easyMAG® and Dx RTS, respectively. It also has good stability: the results of internal variability assay showed that the coefficient of variation ranged between 0.51% and 0.95% for PeV-A1 and between 0.66% and 1.42% for PeV-A2; The results of the inter variability assay showed that the coefficient of variation ranged between 0.85% and 2.08% for HPeV1 and between 1.15% and 2.90% for HPeV2. Fast Track respiratory diagnosis (FTD 33) has good sensitivity with the LOD of 10^3^ copies/mL and has good stability and specificity. At the same time, they have a slight disadvantage: Biomérieux's Biofire (meningo-encephalitis panel) only verifies PeV-A1 and PeV-A2 in specificity tests, rather than other types of PeV-A; The FTD kit can detect PeV-A, but cannot further determine the genotype of PeV-A. (https://www.biomerieux-diagnostics.com/sites/clinic/files/bes_poster_ecv2013.pdf) (https://www.siemens-healthineers.com/molecular-diagnostics/molecular-diagnostics-in-vitro-diagnostics/ftd-respiratory-assays).

When 728 stool samples from healthy children in Beijing, China, were tested using this established real-time PCR method as well as a previous method [17], 11 and eight samples tested positive, respectively. In the process of typing enteroviruses, the *5'-UTR*, *VP4* and *VP2* regions were first chosen for typing, but later it was found that molecular typing based on the *VP1* region was more consistent with serotypes, and since enteroviruses are very similar to PeV-A, genotyping of PeV-A based on the *VP1* region has been used to date [14, 15]. Nested PCR amplification of the *VP1* region was performed on the 11 positive real-time PCR samples, and their *VP1* sequences were successfully obtained using the primer sequences designed in this study. Additionally, EV Genotyping Tool, BLAST, and ML tree results, based on the *VP1* region, showed that of the 11 strains isolated from the stool samples, eight were PeV-A1, one was PeV-A4, and two were PeV-A6. Comparisons also revealed that the nucleic acid-based similarity between the eight PeV-A1 strains and the prototype strain was not very high (73.9–75.4%), and the combination of the results of nucleic acid similarity analysis with the ML tree based on the PeV-A prototype strains indicated that PeV-A1 exhibits a large evolutionary trend in China. In addition, the ML tree, which was established via BLAST analysis, showed that three of the PeV-A1 strains clustered with strains isolated from Belarus, while the remaining five PeV-A1 strains, as well as the PeV-A4 and PeV-A6 strains, clustered with strains isolated from Hong Kong. Thus, a majority of the isolates were endemic. Reportedly, parechoviruses are associated with a wide range of gastrointestinal, respiratory, and central nervous system infections [22]. Although parechovirus infections cannot be treated directly, a rapid and sensitive detection method for their identification may reduce hospitalization and shorten the duration of antibiotic treatment. Therefore, we believe that our study lays the foundation for further research on PeV-As and helps expand the gene sequence information available in GenBank.

This study has some limitations. First, although the *5'-UTR* region is the most stable region relative to other regions, there is the potential for mutations, deletions and gene recombination over time, further leading to off-target probe-primer and false negative results. Second, due to sample reasons, except for PeV-A1, 4, and 6 genotypes, we did not detect other PeV-A genotypes from the 728 stool samples using this method. In addition, although PeV-A1 and PeV-A3 are the most common types in the world, it is indeed a shortcoming of this study to the determination of LODs for only these two types rather than all standards. Finally, we only performed specificity studies on the enterovirus species A, B, and C and did not validate the accuracy of our system using other species. Therefore, we think that the real-time RT-PCR method for the detection of pan-PeV-As can be improved in future.

## Conclusions

In this study, we developed and validated a real-time RT-PCR assay for the identification of PeV-As using 728 clinical samples. This real-time RT-PCR assay showed good reproducibility and specificity, and given that the 5′-*UTR* regions of different types of PeV-As are highly conserved, this assay method possibly has potential for application in the detection of new or emerging PeV-As. In addition, we amplified the *VP1* region of the 11 detected PeV-A genotypes using the primers designed in this study. Typing results indicated that eight, one, and two strains were PeV-A1, PeV-A4, and PeV-A6, respectively. We also presented the results of the genetic characterization and phylogenetic analyses of *VP1* region of these 11 genotype sequences. Therefore, we believe that our study provides a firm foundation for further studies on PeV-As and helps expand the gene sequence information available in GenBank.

## Data Availability

*VP1* genome nucleotide sequences corresponding to the 11 PeV-A strains determined in this study have been deposited in the GenBank nucleotide sequence database under Accession Numbers MW448141–MW448151.

## Abbreviations

EV: Enterovirus
5′-UTR: 5′-Untranslated region
ORF: Open reading frame
3′-UTR: 3′-Untranslated region
LOD: Limit of detection
Ct: Threshold cycle
SD: Standard deviation
CV: Coefficient of variation
R^2^: Coefficient of determination
CDC: Chinese Center for Disease Control and Prevention
